# Reply to Fernández et al. Comment on “Goudra et al. Anesthesia for Bronchoscopy—An Update. *J. Clin. Med.* 2024, *13*, 6471”

**DOI:** 10.3390/jcm14217709

**Published:** 2025-10-30

**Authors:** Basavana Goudra, Lalitha Sundararaman, Prarthna Chandar, Michael Green

**Affiliations:** 1 Department of Anesthesiology and Perioperative Medicine, Thomas Jefferson University, Philadelphia, PA 19107, USA; michael.green2@jefferson.edu; 2 Sidney Kimmel Medical College, 111 S 11th Street, #8280, Philadelphia, PA 19107, USA; prarthna.chandar@jefferson.edu; 3 Department of Anesthesiology, Brigham and Women’s Hospital, 75 Francis St., Boston, MA 02115, USA; 4 Department of Pulmonary, Allergy and Critical Care, Thomas Jefferson University, Philadelphia, PA 19107, USA

Thank you for reading our review and providing valuable feedback [1]. We hope the following addresses your questions.

Performing bronchoscopic procedures, predominantly under general anesthesia, is more of a US preference; both patients and pulmonologists favor this approach. However, we agree that many of these procedures could be performed under deep or even moderate sedation. A motionless patient certainly provides the optimum condition to the bronchoscopist performing the procedure; nevertheless, it is expensive. Often, the choice of anesthesia/sedation is an institutional practice.

Regarding the use of vasopressors, the incidence of intraprocedural hypotension is known to be high with propofol-based intravenous anesthesia. In a recent study, Kotani et al. observed a 21.6% incidence of hypotension at induction with the propofol group (in comparison to the remimazolam group (11.9%)) [2]. They were treated with both ephedrine and phenylephrine, and patients in the propofol group required higher amounts of ephedrine. Hypotension occurs even more frequently in the elderly (59.7% vs. 33.3%) [3]. Depending on the center, in the USA, vasopressor infusions, mainly phenylephrine, are often employed for maintaining blood pressure. It is stated that a mean blood pressure below 60 mm Hg is associated with a decreased hepatic metabolism of drugs, worsening of hypoxemia, and delayed recovery from anesthesia. Rarely, neuromuscular complications and central nervous system abnormalities, including blindness after anesthesia, are possible [4]. Although the cited study included only patients undergoing vascular surgery, Wales et al. concluded that patients who sustained a 40% decrease from the preinduction mean arterial blood pressure with a cumulative duration of more than 30 min suffered more frequent postoperative myocardial injury [5].

In the USA, in the operating/procedure rooms, propofol is always administered by an anesthesia provider. This includes a physician, an anesthesiologist, a certified registered nurse anesthetist, or an anesthesiology assistant. However, administration of moderate sedation (more popularly referred to as conscious sedation) is usually performed by a certified nurse under the supervision of a proceduralist, in this case, the pulmonologist performing the procedure.

We agree that in carefully selected patients, airway adjuncts such as SuperNO_2_VA™ add to the safety of deep sedation. We have used them extensively in patients undergoing gastroenterological procedures, including endoscopic retrograde cholangiopancreatography. However, we have no experience of using it in bronchoscopic procedures.
